# Historical redlining is associated with disparities in wildlife biodiversity in four California cities

**DOI:** 10.1073/pnas.2321441121

**Published:** 2024-06-11

**Authors:** Cesar O. Estien, Mason Fidino, Christine E. Wilkinson, Rachel Morello-Frosch, Christopher J. Schell

**Affiliations:** ^a^Department of Environmental Science, Policy, and Management, University of California, Berkeley, CA 94720; ^b^Department of Conservation and Science, Lincoln Park Zoo, Chicago, IL 60614; ^c^California Academy of Sciences, San Francisco, CA 94118; ^d^School of Public Health, University of California, Berkeley, CA 94720

**Keywords:** redlining, iNaturalist, environmental justice, legacy effects, species richness

## Abstract

Legacy effects describe the persistent, long-term impacts on an ecosystem following the removal of an abiotic or biotic feature. Redlining, a policy that codified racial segregation and disinvestment in minoritized neighborhoods, has produced legacy effects with profound impacts on urban ecosystem structure and health. These legacies have detrimentally impacted public health outcomes, socioeconomic stability, and environmental health. However, the collateral impacts of redlining on wildlife communities are uncertain. Here, we investigated whether faunal biodiversity was associated with redlining. We used home-owner loan corporation (HOLC) maps [grades A (i.e., “best” and “greenlined”), B, C, and D (i.e., “hazardous” and “redlined”)] across four cities in California and contributory science data (iNaturalist) to estimate alpha and beta diversity across six clades (mammals, birds, insects, arachnids, reptiles, and amphibians) as a function of HOLC grade. We found that in greenlined neighborhoods, unique species were detected with less sampling effort, with redlined neighborhoods needing over 8,000 observations to detect the same number of unique species. Historically redlined neighborhoods had lower native and nonnative species richness compared to greenlined neighborhoods across each city, with disparities remaining at the clade level. Further, community composition (i.e., beta diversity) consistently differed among HOLC grades for all cities, including large differences in species assemblage observed between green and redlined neighborhoods. Our work spotlights the lasting effects of social injustices on the community ecology of cities, emphasizing that urban conservation and management efforts must incorporate an antiracist, justice-informed lens to improve biodiversity in urban environments.

## Significance

The legacy of redlining has had dramatic consequences on human and environmental health. Yet, our knowledge of the ecological consequences of redlining on wildlife remains nascent. Using contributory science data, we show that biodiversity is greatly diminished across six taxonomic clades in redlined neighborhoods, including mammals, birds, and insects. We also provide evidence suggesting that unique species are detected with less effort in greenlined than redlined neighborhoods. Thus, policies designed to address biodiversity conservation will greatly benefit from considering land-use legacies and the accompanying societal inequities that impact species resilience, ultimately affecting urban resilience, function, and human health. Our work bolsters the case for integrating social and environmental justice as a critical lens in creating more equitable and biodiverse cities.

Urban biodiversity is quintessential for ecosystem functioning, services, and resilience (1–3), ultimately influencing human and environmental well-being (4). For instance, bottom–up processes are strengthened by high plant diversity providing more ecological niches to support a greater diversity of fauna relative to more species-depauperate areas. Greater plant diversity also mitigates climate-induced challenges by maintaining biogeochemical processes and regulating ecosystem dynamics that support ecological resilience in the face of increasing environmental stochasticity (2, 5). Animal biodiversity can similarly undergird ecosystem function and processing via pollination services and regulating populations, which have myriad positive feedbacks on global food systems and maintaining dynamic species relationships that support more biodiversity (5–7). However, the spatial distribution of urban biodiversity and the environmental components necessary to support urban species are markedly unequal. Thus, determining the factors that generate an unequal distribution of species is essential to strengthening ecosystem resilience.

Cities are structured by systemic racial biases (8), effectively creating resource inequities such as differentiated access to healthcare and education, as well as socioeconomic disparities (9–13). Resource inequity can undergird ecological components, wherein higher socioeconomic neighborhoods have greater biodiversity, commonly referred to as the luxury effect (14). This widespread phenomenon has been noted across taxa, including in avian and mammalian clades (15–17). Additionally, vegetation and canopy cover—which buffer against air pollutants and reduce urban heat island effects—can vary with socioeconomics (18, 19). Thus, societal inequity fundamentally biases resource distributions, shaping differences in environmental quality across cities. To understand the ecology of cities, it is imperative to unpack how the social dimensions of cities influence environmental quality and biodiversity (20, 21).

Redlining—a discriminatory lending practice in the United States institutionalized by the home owner’s loan corporation (HOLC) and supported by the Federal Housing Association (FHA) following the Great Depression—has been shown to influence the environmental quality of urban neighborhoods (22–26). Starting in the 1930s, the HOLC appraisers ranked and mapped neighborhood quality from grade A (i.e., favorable and “greenlined” areas), which were largely composed of high-income and white populations, to grade D (i.e., hazardous and “redlined” areas), which were composed of majority Black and/or minoritized populations (27). These maps reflected neighborhoods that would have been redlined by the FHA and local lenders, serving as a proxy for numerous prior and existing racialized policies at the federal, state, and local level that reinforced racial segregation, discrimination, and disinvestment, both historically and contemporarily, in redlined neighborhoods (28, 29). Historically redlined neighborhoods have thus been associated with poor environmental quality in the present day, with redlined neighborhoods having more environmental hazards such as higher pollution burdens and heat risk than those with higher HOLC grades (30). As a result, redlining has also been associated with adverse human health effects such as preterm births, cancer, and asthma (30, 31), among other health outcomes. This unequal distribution of environmental hazards may be equally salient for wildlife inhabiting cities. Yet, ecologists are just beginning to grasp the potential connections between historical redlining and wildlife ecology (8).

Given the long-lasting impacts of redlining on contemporary environmental quality, it is likely that urban biodiversity may be similarly affected in the United States (8). Indeed, recent work has shown that redlining is associated with the distribution of urban bird biodiversity in Los Angeles (32) and bird biodiversity sampling across the United States (33). However, the linkage between historical redlining and other taxa remains uncertain, and the association between redlining and faunal biodiversity may vary by clade. For example, the life-histories and ecologies of insects and amphibians (e.g., limited movement, smaller home ranges, etc.) may increase these species’ relative exposures to harsh environmental conditions associated with redlining. Consequently, we may observe greater species reductions in certain clades relative to others, such as birds and mammals. Moreover, the association between previously redlined neighborhoods and wildlife may vary across cities due to differences in city size and climate—as seen with income disparities (15). Yet, there is no empirical work that articulates how redlining legacies are differentially experienced across clades and cities.

Examining the association between redlining across multiple clades and cities requires incredibly fine-scale data and large geographic coverage. Contributory science data—where individuals report data voluntarily—can alleviate this due to the vast spatial coverage and low-cost (34, 35). Despite biases within these data (36–39), contributory data sources are incredibly powerful for answering large-scale questions concerning biodiversity. One of the more prominent contributory platforms—iNaturalist—has proven essential for assessing urban biodiversity due to its vast taxonomic coverage (40). Over 40% of the recorded observations that are not from birds in the Global Biodiversity Information Facility (GBIF), the largest global repository of biodiversity data, come solely from iNaturalist, and over 50% of the unique species cataloged in GBIF are derived from iNaturalist (41). Indeed, such contributory data provide extraordinary resolution to understand local to global patterns in species diversity (42), evaluate how urbanization affects biodiversity hotspots (43), and assess species’ responses to climate change (44). Contributory data therefore provide an ideal data source to examine the relationship between historical redlining and urban biodiversity across various cities.

Here, we merged HOLC maps with contributory science data (iNaturalist) to determine whether redlining was associated with differences in faunal biodiversity in Californian cities. We focused on California, the most biodiverse and populous state in the United States, with some of the largest cities co-located with biodiversity hotspots. In addition, our previous work has demonstrated that previously redlined neighborhoods in California have higher pollution burdens, less vegetation, elevated temperatures, and more noise (45)—habitat conditions that likely structure neighborhood biodiversity via bottom–up processes (8). First, we predicted that greenlined neighborhoods would detect more unique species with less sampling effort than redlined neighborhoods due to differences in green space and vegetation as well as potential skews in participation (33, 46). Next, we examined species richness (i.e., alpha diversity) within each HOLC grade and predicted that after controlling for the effect of urbanization (i.e., urban intensity), neighborhood area, and uneven sampling, that redlined neighborhoods would have reduced species richness and native biodiversity relative to greenlined neighborhoods due to reductions in environmental quality (8). Finally, we examined differences in species communities (i.e., beta diversity) by comparing species assemblages among HOLC grades. We predicted that greenlined neighborhoods would be more dissimilar to redlined neighborhoods due to strong differences in environmental quality (8, 30, 45). We expected that HOLC grades that were closely ranked (i.e., A vs. B or C vs. D) would not differ in species assemblages.

## Results

### Accumulated Species Richness.

We calculated accumulated species richness, i.e., cumulative observed species richness, by correlating the observed number of unique species with the number of total observations across all species per HOLC grade. We extracted accumulated species richness in greenlined neighborhoods and the observations needed to reach this total. We then used this value to calculate the differences in observations needed for redlined neighborhoods to reach an equivalent accumulated species richness in greenlined neighborhoods. Accumulated species richness deals with biases in biodiversity sampling, which can contribute to differences in observed biodiversity based on observations within a HOLC grade and is crucial for equitable conservation.

**Fig. 1.**
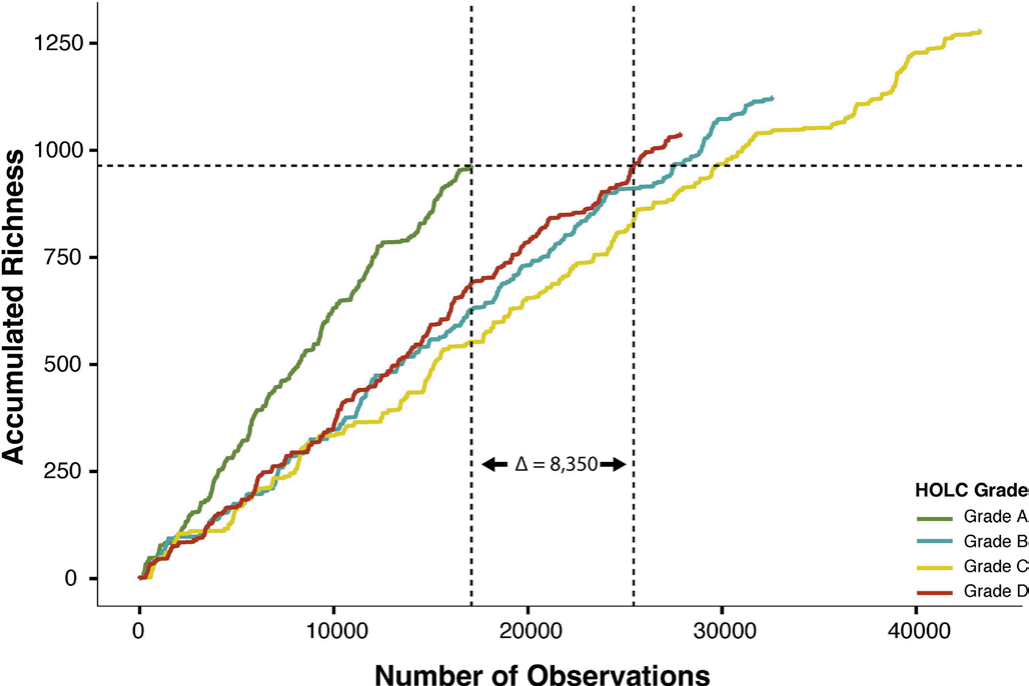
Greenlined neighborhoods detect more unique species with less sampling effort. Species accumulation curves for each HOLC grade across six clades. The x-axis shows the number of observations within each HOLC grade. The y-axis shows accumulated species richness. The dashed horizontal line* shows the maximum accumulated richness for grade A. The vertical lines** show the number of observations to reach grade A’s maximum accumulated richness in grade A (left vertical line) and in grade D (right vertical line). The difference in observations between redlined (i.e., grade D) and greenlined (i.e., grade A) neighborhoods is shown as a delta value. *Horizontal line: y = 964. **Vertical lines (grade A, grade D): x = 17,095; 25,445.

We found that grade C had the highest accumulated species richness (1,281 species), followed by B (1,124 species), D (1,039 species), and A (964 species) (Fig. 1). In grade A, it took 17,095 observations to reach the grade’s maximum observed species richness (964). To reach an observed species richness of 964 in grade D, it took 25,445 observations (Δ = 8,350), while in grades B and C, it took 27,519 (Δ = 10,424) and 29,730 (Δ = 12,635) observations, respectively (Fig. 1). We observed this trend between grades A and D for all cities except for San Diego, where grade D reached the maximum species richness observed in grade A with fewer observations (*SI Appendix*, Fig. S1). We also observed this trend in accumulation curves for native and nonnative species, though the delta values between grades A and D were smaller (native Δ = 621; nonnative Δ = 691) (*SI Appendix*, Fig. S2).

### Alpha Diversity: Species Richness.

We analyzed species richness across six clades: birds, mammals, insects, arachnids, reptiles, and amphibians. We used a Bayesian approach to parameterize our model with HOLC grade and percentage of impervious surface as fixed effects. The model intercept and HOLC grade were also allowed to vary by city (i.e., random intercept and slope terms) to quantify associations between HOLC grades and species richness across cities. We included area and the number of observations as a log-offset term to control for differences in neighborhood size and sampling effort (*Materials and Methods*). We used this model to predict species richness for each neighborhood rather than raw data to control for uneven sampling (Fig. 1). We then disaggregated our data into native and nonnative species to ascertain potential drivers of overall species richness. For overall, native, and nonnative species richness, we found significant differences among HOLC grades (Table 1).

**Table 1.** Species richness across HOLC grades

City	HOLC grade	Mean richness	Mean native richness	Mean nonnative richness
ALL	A	20.4 (3.25, 65.29)	12.72 (2.06, 40.82)	7.47 (1.10, 24.73)
ALL	B	14.65 (1.60, 92.26)	8.9 (0.98, 56.53)	5.81 (0.60, 36.69)
ALL	C	11.74 (1.45, 62.93)	7.5 (0.95, 39.07)	4.20 (0.47, 24.22)
ALL	D	5.57 (0.72, 28.32)	3.39 (0.46, 16.99)	2.17 (0.25, 12.33)
Los Angeles	A	12.15 (11.65, 12.65)	7.16 (6.79, 7.55)	5.2 (4.85, 5.58)
Los Angeles	B	24.17 (23.55, 24.8)	14.71 (14.23, 15.21)	9.69 (9.29, 10.1)
Los Angeles	C	10.78 (10.57, 10.98)	6.83 (6.66, 7)	4.09 (3.97, 4.22)
Los Angeles	D	5.99 (5.81, 6.17)	3.57 (3.43, 3.71)	2.53 (2.41, 2.65)
Oakland	A	10.3 (9.56, 11.10)	6.71 (6.11, 7.34)	3.25 (2.83, 3.71)
Oakland	B	20.66 (19.65, 21.70)	12.29 (11.54, 13.07)	8.24 (7.56, 8.95)
Oakland	C	18.02 (17.31, 18.75)	10.89 (10.36, 11.44)	6.77 (6.32, 7.25)
Oakland	D	5.23 (4.94, 5.53)	3.06 (2.85, 3.27)	2.11 (1.92, 2.31)
San Diego	A	48.73 (45.43, 51.98)	32.56 (29.98, 35.23)	16.33 (14.36, 18.47)
San Diego	B	23.55 (22.50, 24.64)	15.81 (14.95, 16.7)	7.49 (6.91, 8.1)
San Diego	C	12.81 (12.16, 13.49)	8.95 (8.38, 9.54)	3.73 (3.42, 4.06)
San Diego	D	4.49 (4.21, 4.79)	3.2 (2.95, 9.54)	1.24 (1.11, 1.38)
San Francisco	A	35.84 (32.02, 39.97)	20.77 (18.19, 23.49)	10.4 (8.28, 12.87)
San Francisco	B	4.9 (4.64, 5.16)	2.63 (2.46, 2.8)	1.83 (1.66, 2.01)
San Francisco	C	9.3 (8.83, 9.79)	5.63 (5.28, 6)	2.57 (2.35, 2.8)
San Francisco	D	6.86 (6.42, 7.31)	3.77 (3.48, 4.07)	2.34 (2.08, 2.61)

**Fig. 2.**
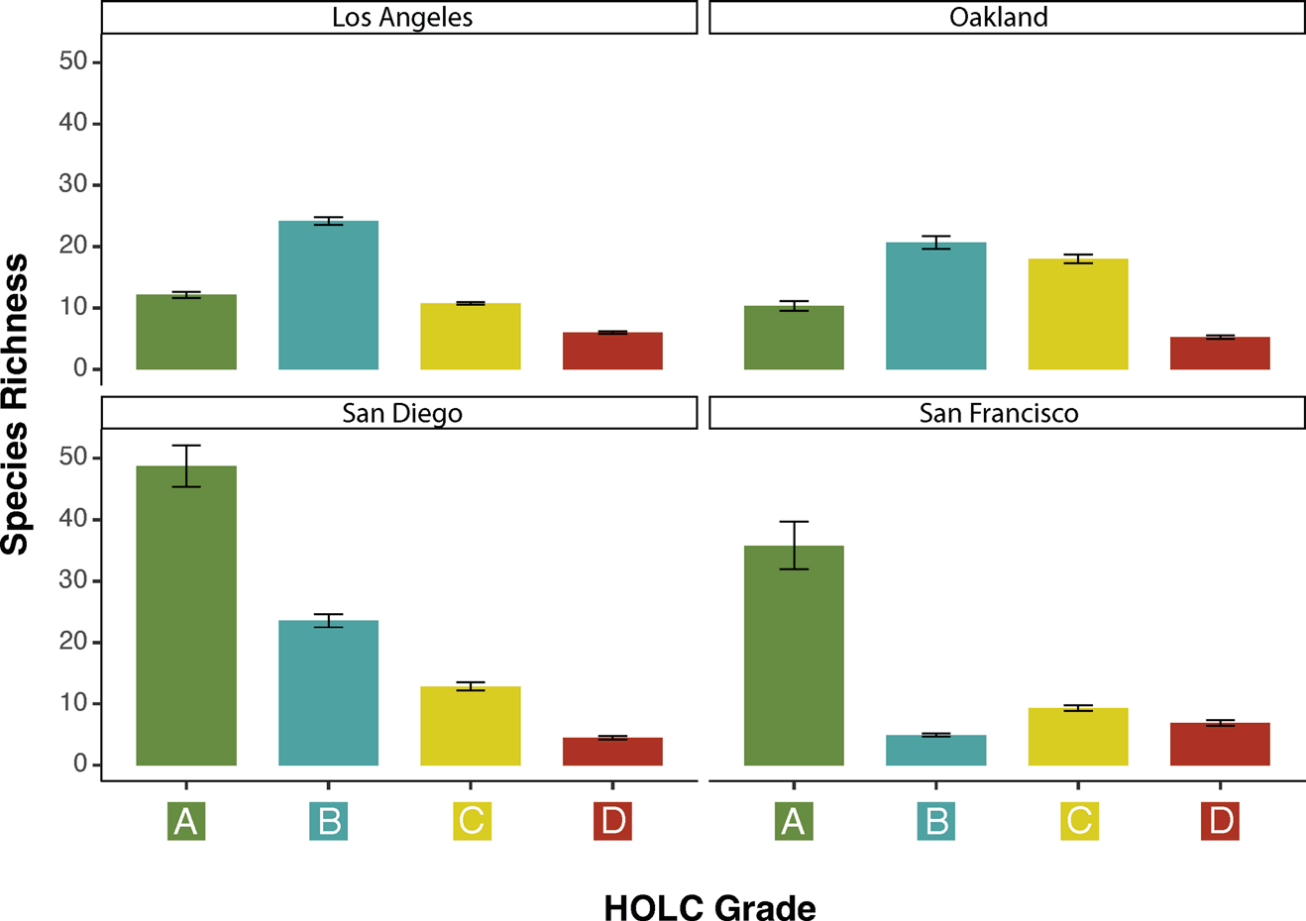
Redlined neighborhoods across California have lower species richness. Species richness for all species across six clades among HOLC grades for Los Angeles (*Top Left*), Oakland (*Top Right*), (C) San Diego (*Bottom Left*), and San Francisco (*Bottom Right*). Bars represent the mean, and whiskers represent 95% credible intervals. All pairwise comparisons are significant.

After controlling for urban intensity, neighborhood area, and the number of observations in a HOLC neighborhood, we found that California redlined neighborhoods had the lowest species richness (Fig. 2 and Table 1), including at the clade-level (*SI Appendix*, *Supporting Information 1* and Table S1). On average, across all cities, grade A had the highest species richness (median = 20.41, CI: 3.18, 65.15), followed by grades B (14.61, CI: 1.59, 92.33), C (11.82, CI: 1.43, 62.72), and D (5.59, CI: 0.71, 28.88), with significant differences between grades A and D (median: 23.95, CI: 0.80, 47.11) (Table 1). Similar trends were observed for native species richness, with redlined neighborhoods having the lowest native species richness (3.39, CI: 0.46, 16.99) and significant differences between grades A and D (15.07, CI: 0.61, 29.53) (Table 1). We found no significant differences among HOLC grades for nonnative species richness.

We found significant differences between each HOLC grade per city. However, cities varied in how HOLC grades were associated with species richness with redlined neighborhoods holding the lowest species richness in three of the four cities examined (Fig. 2 and Table 1). San Diego and San Francisco had the largest disparities in average species richness between greenlined and redlined neighborhoods, at Δ = 44 and Δ = 29, respectively, compared to Los Angeles (Δ = 6) and Oakland (Δ = 5). Species richness trends did not always follow the ranked HOLC grading at the city level (Fig. 2). While San Diego followed the ordered trend, San Francisco, Los Angeles, and Oakland had different patterns. In San Francisco, greenlined neighborhoods had the highest species richness and were followed by grades C, D, and B. In Los Angeles, B-grade neighborhoods had the highest species richness, followed by grades A, C, and D, and similarly, in Oakland, B-grade neighborhoods had the highest species richness but were followed by grades C, A, and D (Table 1). Similar trends were observed for native and nonnative richness (Table 1 and *SI Appendix*, Figs. S3 and S4). We found significant differences at the city level between most HOLC grades for native and nonnative species richness except native richness in Los Angeles between grades A and C (0.34, CI: −0.10, 0.78) and nonnative richness in San Francisco between grades C and D (0.23, CI: −0.12, 0.57).

**Fig. 3.**
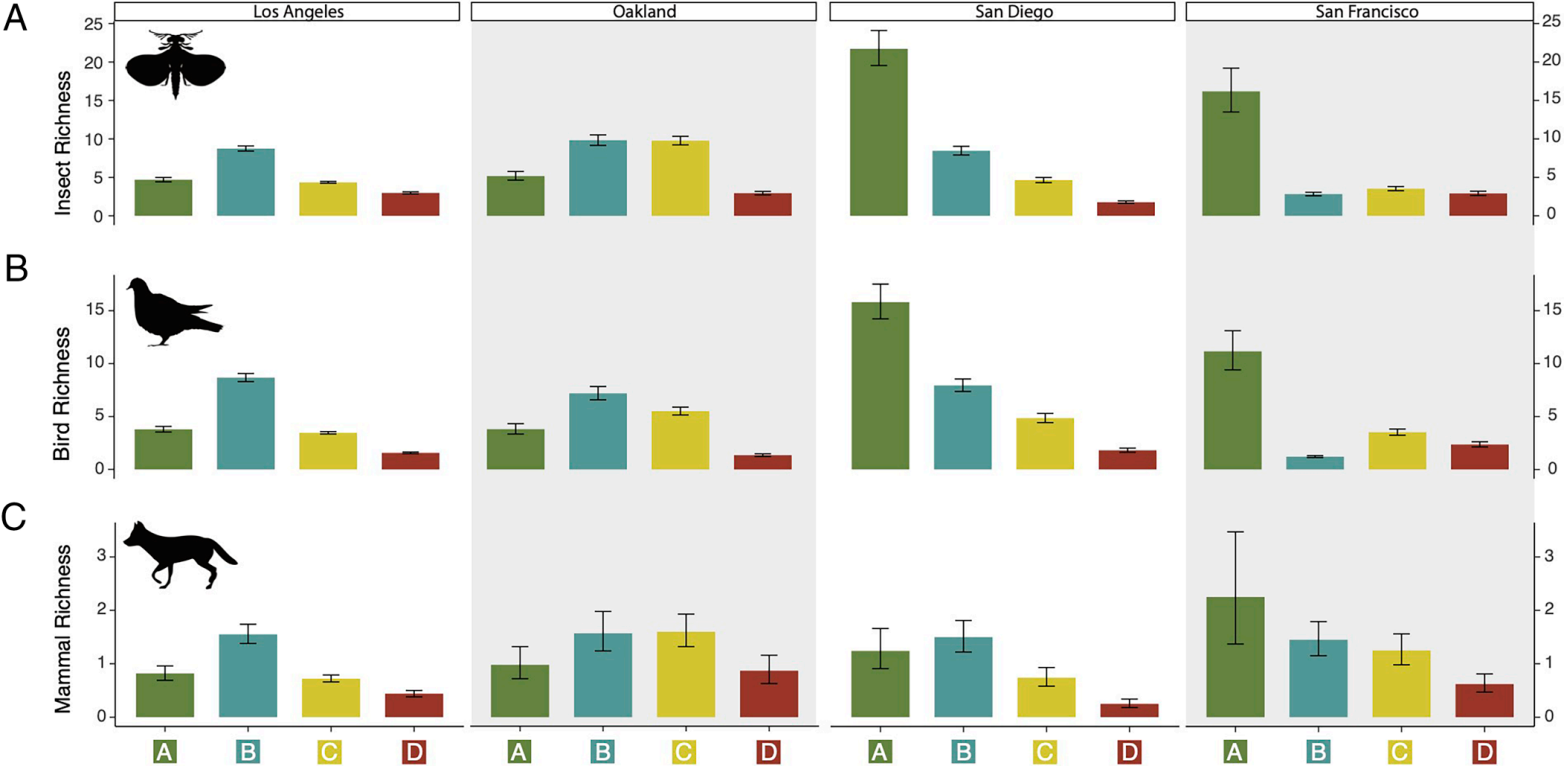
Clade richness is consistently lower in redlined neighborhoods. Species richness for (*A*) insects (top row), (*B*) birds (middle row), and (*C*) mammals (bottom row) shown among HOLC grades for each Californian city (columns). Los Angeles is on the far left, Oakland is on the middle left, San Diego is on the middle right, and San Francisco is on the far right. Bars represent the mean, and whiskers represent 95% credible intervals. All pairwise comparisons are significant. All comparisons between green- (i.e., grade A) and redlined (i.e., grade D) neighborhoods are significant. Note: for each clade, the y-axis (species richness) is subject to change.

We found similar differences in species richness across all six clades, with grades A and B having the highest species richness and grade D having the lowest across clades, except for bird and insect richness in San Francisco, which was slightly lower in grade B (Fig. 3 *A* and *B* and *SI Appendix*, *Supporting Information 1* and Tables S2–S7). We found significant differences at the clade level between green and redlined neighborhoods across all clades within each city, except for mammals, reptiles, and arachnids in Oakland (Fig. 3*C* and *SI Appendix*, Tables S2–S7). We found consistent disparities between green and redlined neighborhoods for native and nonnative richness across clades, though there was some variation (*SI Appendix*, Tables S2–S7).

### Beta Diversity.

**Fig. 4.**
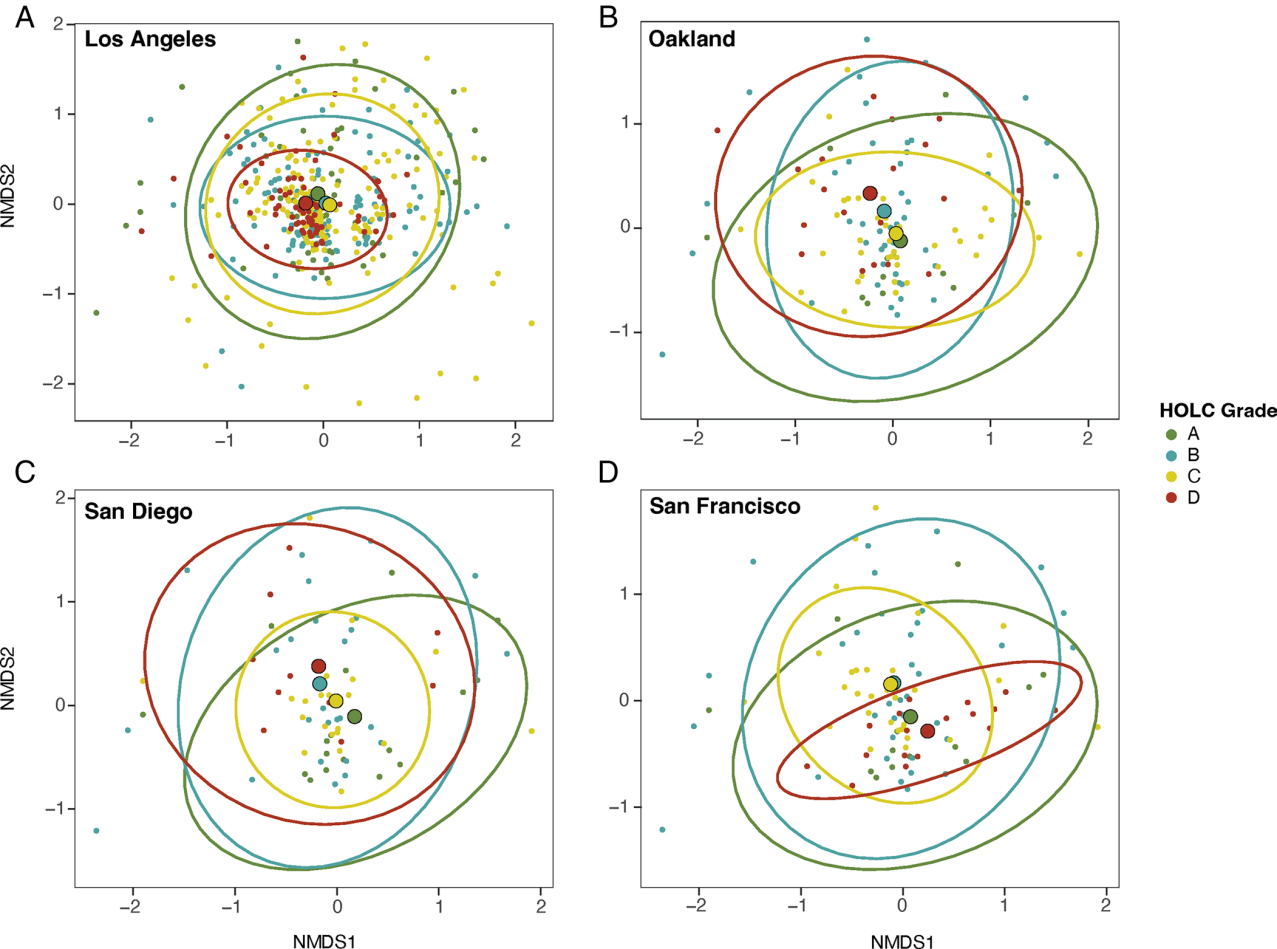
Redlined neighborhoods differ in their species assemblage. Nonmetric multidimensional scaling (NMDS) for β-diversity (Jaccard’s metric) among HOLC grades in (*A*) Los Angeles, (*B*) Oakland, (*C*) San Diego, and (*D*) San Francisco. Each dot represents a neighborhood and ellipses encompass 95% data points. No overlap between ellipses suggests that HOLC grades have distinct beta diversity patterns and strong dissimilarity in species assemblage. Substantial overlap in ellipses suggests that beta diversity between HOLC grades is more similar to each other and there is strong similarity in species assemblage.

We calculated beta diversity (i.e., differences in types of species) using Jaccard’s index and tested for differences in species assemblage among HOLC grades using PERMANOVAs. We found a significant effect of city (R^2^ = 0.0606, F = 15.1827, *P* < 0.0001) and HOLC grade (R^2^ = 0.0091, F = 2.2888, *P* < 0.0001) on beta diversity (Fig. 4 and *SI Appendix*, Figs. S5–S9). We found similar results when we solely examined native (city: R^2^ = 0.0651, F = 16.3574, *P* < 0.0001; HOLC grade: R^2^ = 0.0090, F = 2.2969, *P* < 0.0001), and nonnative species (city: R^2^ = 0.05581, F = 13.6553, *P* < 0.0001; HOLC grade: R^2^ = 0.00939, F = 2.2694, *P* < 0.0001).

Across each city, we found that HOLC grades were associated with beta diversity (Fig. 4 and *SI Appendix*, Table S8). For Los Angeles, we found significant differences in beta diversity between all HOLC grades (*SI Appendix*, Table S8), with grades A and C, A and D, and B and D showing the strongest differences in species assemblages (*P* < 0.001). In San Francisco, we found significant differences between grades A and C (*P* < 0.01), A and D (*P* < 0.001), B and D (*P* < 0.001), as well as B and C (*P* < 0.01). In Oakland, we found significant differences between grades A and D, B and C, and B and D (*P* < 0.05). In San Diego, we found significant differences between grades A and C as well as A and D (*P* < 0.05). For native and nonnative species, we found nearly identical patterns in significant differences between HOLC grades for each city (*SI Appendix*, Figs. S7 and S8 and Table S8), except for native species in San Diego, where no significant differences in beta diversity were found (*P* = 0.1955).

## Discussion

By analyzing 708 previously HOLC-graded neighborhoods in four California cities, we found three main linkages between redlining and biodiversity. First, we found that greenlined neighborhoods detected more unique species in significantly fewer observations than other HOLC grades. Second, redlining was uniformly associated with decreased alpha diversity across all cities and observed in each clade. Third, we found that species assemblages varied in each HOLC grade, with green and redlined neighborhoods having significantly different species assemblages in each city examined. Our work fills a critical empirical gap in the field of urban ecology by examining wildlife biodiversity across a wide variety of animal clades for multiple cities in relation to historical redlining. This extends our understanding of how redlining may be associated with wildlife ecology and biodiversity, which has only recently been highlighted with bird biodiversity in Los Angeles (32). The evidence presented here supports previous theoretical linkages between redlining and faunal biodiversity introduced by Schell et al. (8) across major taxonomic clades, highlighting the connections among systemic racism and urban ecosystems. Disentangling the relationship between redlining and biodiversity provides a critical first step in evaluating how racialized policies have downstream consequences for the community ecology of cities.

In support of our hypotheses, the number of unique species potentially present in a community pool was estimated in fewer observations for greenlined neighborhoods relative to other grades. These results align with recent work by Ellis-Soto et al. (33), showing that bird biodiversity sampling is typically more even and higher in greenlined neighborhoods than in redlined neighborhoods. However, for iNaturalist data in California, this disparity is not due to differences in observation efforts. Our results indicated that nongreenlined neighborhoods (i.e., B, C, and D) had higher observations than greenlined neighborhoods. Rather, our results suggest that an individual is more likely to encounter a greater diversity of species with less search effort in California’s greenlined neighborhoods than in nongreenlined neighborhoods. This may potentially be explained by greenlined neighborhoods customarily having increased environmental quality [i.e., higher vegetation cover and reduced ecological disturbances (24, 30, 45, 47)], which in turn improves the likelihood of unique species occupying the given area. This holds broad implications for human well-being in urban spaces, as equity in nature access and quality, as well as promoting positive human-environment relationships (which are more likely with increased access to biodiverse spaces) are increasingly being considered as central issues of environmental justice (48, 49).

After controlling for differences in observations, neighborhood area, and urban intensity, we found that redlined neighborhoods had less species richness than greenlined neighborhoods. Conversely, there was slight variation in which grade had the highest richness, with greenlined neighborhoods in San Diego and San Francisco and B-grade neighborhoods in LA and Oakland having the highest species richness. Both A and B-graded neighborhoods in California have relatively high environmental quality (45), suggesting that there may be more viable wildlife habitat compared to C and D-graded neighborhoods. We found consistent disparities on the clade level, with redlined neighborhoods frequently exhibiting the lowest species richness. Taken together, our findings indicate that redlining and associated racialized policies, as captured by HOLC maps, have pronounced legacy effects on species richness in spite of apparent social and ecological variation among cities. Further, despite differences in mobility and tolerance to environmental hazards associated with redlining, our evidence suggests wildlife across all clades are detrimentally impacted by the legacy effects of redlining. The legacy effect of redlining is particularly pronounced in San Diego and San Francisco, with large differences in species richness between green and redlined neighborhoods across most taxonomic groups. This may be due in part to differences in street tree and local gardens availability and distribution as well as greenspace area, quality, and size throughout these cities. Street trees and local gardens can offer food sources and simultaneously serve as refugia and habitat for wildlife, including birds and insects, thereby promoting local biodiversity (50–53). In tandem, urban greenspaces can often function as de facto biogeographic “islands,” with larger and closer patches showing increased species richness (54, 55). Moreover, habitat complexity throughout urban neighborhoods, including parks, such as mixed vegetation types, understory vegetation availability, and community gardens, can support neighborhood biodiversity via constructing more diverse ecological niches (53, 55–58). Thus, it is possible that compared to Los Angeles and Oakland, the disparities in habitat complexity, including the amount of street trees, understory vegetation, and general flora biodiversity, between green and redlined neighborhoods are greater in San Diego and San Francisco.

Contrary to our predictions, we failed to detect higher nonnative species richness in redlined areas. Rather, we found that greenlined neighborhoods had higher nonnative species than nongreenlined neighborhoods. Greenlined neighborhoods may have higher nonnative species richness for several reasons. While urban areas generally have high levels of nonnative species (59), they are not uniformly distributed. High-income urban neighborhoods tend to have higher abundances of nonnative trees and plants (60, 61), which have the potential to dampen native richness by selecting against species that may rely on native plants (62, 63). A reduction of native species can free up space within an ecosystem, potentially allowing for nonnative species to spread and establish within an environment (64). Moreover, the intentional selection of nonnative plant species with desirable aesthetic characteristics (e.g., flowering, ornamentation, and color) can bolster their abundance in greenlined neighborhoods (65). Nonnative plants considered aesthetically pleasing are often expensive, and residents who live in greenlined areas often have more economic mobility to purchase these plants. Thus, although nonnative species tend to do better in more disturbed habitats (66, 67), such as redlined neighborhoods, our results suggest that varying social–ecological drivers of plant communities across neighborhoods may dilute or offset any potential differences in nonnative species richness across red and greenlined areas in California.

We found that beta diversity differed between HOLC grades across cities, with green- and redlined neighborhoods having consistently different species assemblages. This result held true when we examined native and nonnative species for each city, except for San Diego, where native species assemblage did not differ across HOLC grades. These results may be explained by city-level attributes that exacerbate the influence of redlining on species assemblages across the observed cities. For instance, San Francisco is California’s most densely populated city (7,194 people/km^2^), with extensive impervious surface cover on a peninsula with a large highway on the southern border. Thus, San Francisco may function as an urban island with limited immigration pathways for terrestrial organisms to colonize the city. In addition, the geographic space is further partitioned by highways I-280 and I-101 in the east, creating multiple urban islands on the peninsula. These biogeographic factors, combined with uneven vegetation, may amplify differences in species pools between greenlined neighborhoods in the west and redlined neighborhoods in the east. Thus, species in San Francisco’s redlined neighborhoods may consist of a few generalist species (e.g., raccoons, pigeons, brown rats, etc.) that can cope with myriad human-driven ecological disturbances. This alteration of species assemblages due to redlining’s influence on ecological characteristics holds implications for the varying degrees of ecosystem services provided by faunal biodiversity at the neighborhood level. These ecosystem services, such as pollinator services or pest regulation, are critical for local justice initiatives (e.g., food sovereignty) as well as overall ecosystem health.

The environmental and societal inequities seen today in neighborhoods are a by-product of racialized policies and practices that have shaped contemporary housing, labor, and economic opportunity (26, 30, 48, 68–72). Moreover, uneven governmental representation due to limited civic action furthers these inequities via racialized zoning, development, and bias in place-based resource investment (72–76). HOLC’s reflection and institutionalization of redlining via creating risk assessment maps captures these and many other discriminatory policies that have ultimately concentrated Black and other minoritized populations, alongside environmental hazards, into redlined neighborhoods (29, 77). Despite HOLC being dissolved in 1951 and redlining made illegal in 1968, racialized zoning, covenants, violence, and steering continued to reinforce the racial distribution reflected in the notes of HOLC maps across the United States (77, 78). Even a century after the creation of the HOLC maps, redlined neighborhoods in the cities we examined continue to face disproportionately high environmental hazards (45). Hence, societal and environmental injustices as a result of racialized policies are associated with variation in local biodiversity in California cities.

Cities have historically been excluded from biodiversity conservation efforts due to this broad assumption that cities represent “biological deserts” devoid of unique species and assemblages (79–81). As the world continues to urbanize, this archaic worldview is becoming less common (80, 82–85). Recent efforts to conserve global biodiversity, such as 30 × 30 and the United Nations’ Goal 15 (86, 87), are now acknowledging cities as important conservation hubs, especially given that more than 400 cities globally are situated in biodiversity hotspots (88, 89). Reimagining cities as biodiversity centers subsequently shifts the focus to assessing the social–ecological drivers that facilitate or hinder species persistence. Our results highlight that societally driven disparities in housing have profound impacts on urban faunal biodiversity in California cities, with redlined neighborhoods having significantly less faunal biodiversity than greenlined neighborhoods. In cities, societal injustices that contribute to disparities in environmental and human well-being are often highly concentrated in marginalized communities (90); thus, urban areas may serve as ideal test cases for understanding the broader impacts of inequities on wildlife via metrics such as biodiversity. Our results demonstrating the association between redlining and faunal biodiversity within and across cities provide a unique set of metrics to bolster ongoing efforts to rectify harmful legacy effects (e.g., City of Oakland’s Race and Equity Department), especially as redlined neighborhoods in California are predominantly composed of marginalized populations along both race and class lines (91). Recognizing and prioritizing social justice will be key for accomplishing equitable conservation and achieving lasting outcomes that safeguard our urban ecosystems for generations.

## Materials and Methods

### Study Region.

Our study takes place throughout the state of California within the United States of America. Within California, eight cities have digitized HOLC maps via the University of Richmond’s Mapping Inequality project (92): Fresno, Los Angeles, Oakland, Sacramento, San Diego, San Francisco, San Jose, and Stockton. In our analysis, we only included cities with at least five observations in each HOLC grade per clade. Thus, our analysis was restricted to Los Angeles, Oakland, San Diego, and San Francisco. Note: the Oakland HOLC map includes Oakland, Berkeley, San Leandro, Piedmont, Emeryville, and Albany and the Los Angeles HOLC map includes the greater Los Angeles area (92).

### Datasets and Geospatial Processing.

We used three data sources: 1) HOLC grade maps via the Mapping Inequality project (92), 2) iNaturalist data, and 3) the National Land Cover Database’s (NLCD) 2019 impervious surface layer.

We downloaded the digitized HOLC maps of California from the Mapping Inequality database and all iNaturalist research-grade observations of mammals, birds, reptiles, amphibians, insects, and arachnids from the past 5 y (January 1, 2017 to January 1, 2022) within HOLC polygons for each city. We selected these years to coincide with the rise in the use of iNaturalist (93), which resulted in 123,235 total observations. Although we selected research-grade observations (94), some rows lacked species information (<50). These rows were filtered out, yielding 123,191 observations. We then filtered the data for the four cities in our analysis, yielding 114,711 observations for biodiversity analysis (1,800 unique species). Because we were interested in differences between native and nonnative species among grades, we then redownloaded native species data from iNaturalist and filtered for native species (via selecting “no” on introduced species and “yes” on native species). We used these data to extract the number of native and nonnative species in our original data frame. To control for differences in observations within our dataset (*SI Appendix*, Fig. S9), we log-offset the number of observations per neighborhood (see details below). Finally, we obtained the mean percentage of impervious surfaces (i.e., urban intensity) from the NLCD for each HOLC neighborhood using Zonal Statistics in ArcGIS Pro.

### Data Analysis.

We investigated the influence of HOLC grade on biodiversity. All statistical analyses were completed in R v.4.1.0 (95) and all plots were made using the ggplot2 package (96).

For biodiversity data, we calculated alpha and beta diversity. For alpha diversity, we calculated the accumulated observed species richness, i.e., the number of unique species in relation to observations within a HOLC grade, and species richness, i.e., the number of unique species. To calculate accumulated observed species richness, we manipulated our data to track the number of observations of species in a HOLC grade as well as the absence of observations (i.e., a value of 0). Hence, a value of 0 does not contribute to species richness but contributes to the observation count, and a value of 1 or higher contributes to species richness and observation count. We used this to visualize how species richness accumulates as observations increase until maximum observed richness is reached in a grade. We calculated beta diversity by using a presence–absence (Jaccard’s) metric in the adonis function via the vegan package (97), which generates values between 0, representing complete dissimilar species assemblages, and 1, representing completely similar assemblages. To examine significant differences in beta diversity among HOLC grades, we used a PERMANOVA with 10,000 permutations and a Benjamin–Hochberg correction.

We used a Bayesian framework to understand the influence of HOLC grade on species richness, using a Poisson mixed-effects model via the nimble package in R (98). Our response variable was the number of species observed in each HOLC neighborhood. We included HOLC grade as a fixed effect, though the model intercept and HOLC grades were allowed to vary by city (i.e., a random intercept, random slope model). To account for variation in sampling and neighborhood size, we logged and summed neighborhood area and number of observations per neighborhood, which we then included as an offset term in the model. Before log-offsetting observations, we ensured that each neighborhood had at least one observation (99). Finally, we included impervious surfaces in our model to control for the negative influence urbanization has on species richness (15, 100, 101). We did not include vegetation in our model as vegetation (i.e., normalized difference vegetation index) and impervious cover are strongly and negatively correlated (Pearson correlation coefficient for all cities: −0.8982; Los Angeles: −0.9271; Oakland: −0.9626; San Diego: −0.9199; San Francisco: −0.8899). Thus, although our analysis does not control for vegetation explicitly, it does control for a variable that strongly covaries with it.

In our model, fixed effects were given Normal (0, 2) priors, while SD terms associated with city-level random effects were given Gamma (1,1) priors. Following a 110,000-step burn-in, we sampled the posterior for 40,000 iterations across 4 chains. To check for model convergence, we ensured that Gelman–Rubin diagnostics were <1.10 (102). To examine whether there were significant differences between HOLC grades, we conducted hypothesis testing in a Bayesian framework. After fitting our model, we calculated contrasts between each HOLC grade, representing differences in species richness between grades. We then calculated the credible intervals of these differences and examined whether they overlapped zero. For significant differences, we report the median and CIs.

## Supplementary Material

Appendix 01 (PDF)

## Data Availability

All data used in this manuscript has been deposited at (103).
