# Supplementary material for: Historical redlining is associated with disparities in wildlife biodiversity in four California cities
Source: Proc Natl Acad Sci U S A. 2024 Jun 11;121(25):e2321441121. doi: 10.1073/pnas.2321441121 (PMC11194601; doi:10.1073/pnas.2321441121)
Supplement: Supplementary file 1 — Appendix 01 (PDF) [file pnas.2321441121.sapp.pdf]

Supporting Information For

Historical redlining is associated with disparities in wildlife biodiversity in four California cities

Cesar O. Estien*, Mason Fidino, Christine E. Wilkinson, Rachel Morello-Frosch, and Christopher J. Schell

*corresponding author: Cesar O. Estien
Email: cestien@berkeley.edu

This PDF file inlcudes:

Additional Results

Figures S1-S9

Table S1-S8

Supporting Information 1: Results

Clade-level species richness across HOLC grades

Statewide

Across clades, we found variation in species richness across HOLC grades for all, native, and nonnative species (Table S2-7). For insects, we found that greenlined neighborhoods had the highest species richness on average but found no significant differences between grades. We found the same trends for native and nonnative insect richness. For arachnids, we found that B-graded neighborhoods had the highest species richness on average but found no significant differences between grades. We found the same trends for native and nonnative arachnid richness. For birds, we found that greenlined neighborhoods had the highest species richness on average and found significant differences between green and redlined neighborhoods (8.10, CI: 0.63, 15.57). We found similar trends in native bird richness, but not nonnative bird richness between green and redlined neighborhoods (1.13, CI: -0.39, 3.07). For mammals, we found that B-grade neighborhoods had the highest species richness on average but found no significant differences between grades. We found similar trends in native and nonnative mammalian richness but found significant differences between grades B and D (1.77, CI: 0.07, 3.47). For reptiles, we found that B-grade neighborhoods had the highest species richness on average but found no significant differences between grades. We found similar trends for native and nonnative richness but found significant differences in nonnative reptiles between grades B and D (1.74, CI: 0.03, 3.45). For amphibians, we found that B-grade neighborhoods had the highest species richness on average, and we found significant differences between grades A and C (0.48, CI: 0.04, 0.92), A and D (0.73, CI: 0.23, 1.23), and B and D (1.21, CI: 0.05, 2.37). We found similar trends for native richness, except no significant differences between A and C were found (0.43, CI: -0.07, 0.94). We found no significant differences in nonnative amphibian richness between HOLC grades.

City-level

For insects, we found significant differences in richness between green and redlined neighborhoods in every city (Figure 3), and this remained true for native and nonnative insects. For arachnids, we found significant differences in richness between green and redlined neighborhoods in each city except Oakland (-0.11, CI: -0.38, 0.15). For native arachnid richness, we found significant differences between green and redlined neighborhoods in San Diego (2.35, CI: 1.11, 3.59) and San Francisco (1.44, CI: 0.34, 2.53), but not Los Angeles (0.11, CI: -0.04, 0.25) and Oakland -0.09, CI: -0.21, 0.04). For nonnative arachnid richness, we found significant differences between green and redlined neighborhoods in San Diego (3.11, CI: 1.71, 4.51) and Los Angeles (0.93, CI: 0.62, 1.23), but not San Francisco (median = 1.14, CI: -0.19, 2.47) and Oakland (0.08, CI: -0.22, 0.38). For birds, we found significant differences in richness between green and redlined neighborhoods in each city (Figure 3), and this remained true for native and nonnative birds, except for nonnative birds in Oakland (0.13, CI: -0.11, 0.59). For mammals, we found significant differences between green and redlined neighborhoods in each city except for Oakland (0.12, CI: -0.29, 0.54) (Figure 3). These patterns remained true for native and non–native mammals, except for in nonnative mammalian richness in San Francisco (0.66, CI: -0.15, 1.47). For reptiles, we found significant differences between green and redlined neighborhoods

in each city except for Oakland (0.12, CI: -0.23, 0.11), and these trends remained true for native reptiles. For nonnative reptiles, we only found a significant difference between green and redlined neighborhoods in Los Angeles (0.27, CI: 0.03, 0.51) and San Diego (0.47, CI: 0.01, 0.93), but not Oakland (.11, CI: -0.22, 0.44) or San Francisco (0.27, CI: -0.65, 1.18). For amphibians, we found significant differences between green and redlined neighborhoods in each city, and these trends remained true for native and nonnative amphibian richness, except for nonnative amphibians in Oakland (1.51, CI: -2.31, 5.33) and San Francisco (0.22, CI: -0.08, 0.53).

Supporting Information 2: Figures

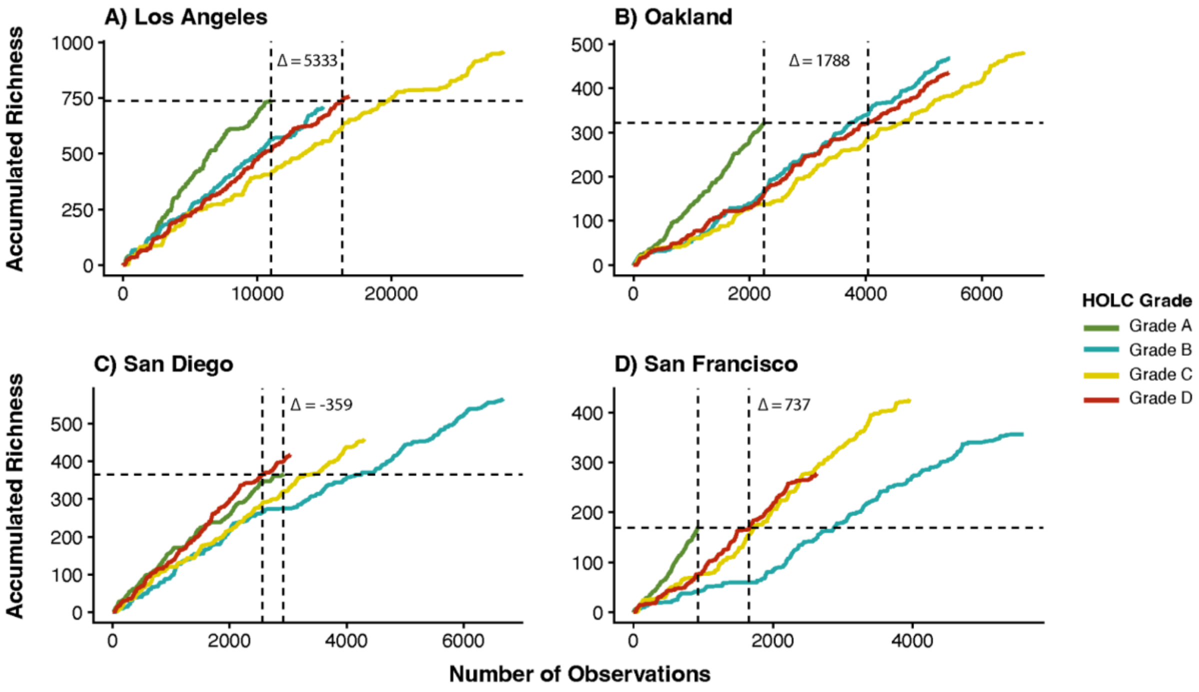

Figure S1. City-level species accumulation curve per HOLC grade. Species accumulation curves for each HOLC grade across six clades for all species in (A) Los Angeles, (B) Oakland, (C) Oakland, and San Francisco. The x-axis shows the number of observations within each HOLC grade. The y-axis shows accumulated species richness. The dashed horizontal line* shows the maximum accumulated richness for Grade A. The vertical lines** show the number of observations to reach Grade A's maximum accumulated richness in Grade A (left vertical line) and in Grade D (right vertical line). The difference in observations between redlined (i.e., grade D) and greenlined (i.e., grade A) and neighborhoods is shown as a delta value.

*Horizontal line (y): Los Angeles: 738; Oakland: 322; San Diego: 365; San Francisco: 169

**Vertical lines (x; grade A, grade D): Los Angeles: 11005, 16338; Oakland: 2247, 4035; San Diego: 2917, 2558; San Francisco: 921, 1658

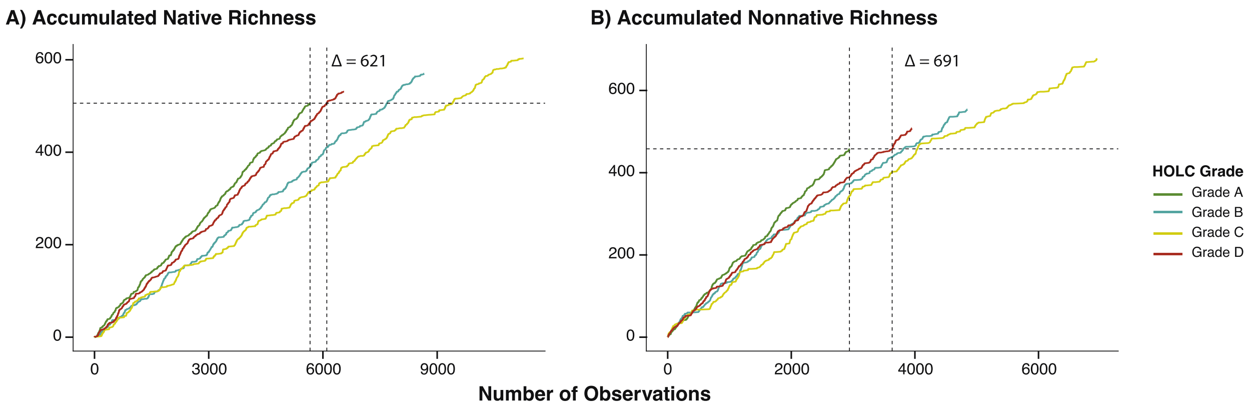

Figure S2. Native and nonnative species accumulation curve per HOLC grade. Species accumulation curves for each HOLC grade across six clades for (A) native species and (B) nonnative species. The x-axis shows the number of observations within each HOLC grade. The y-axis shows accumulated species richness. The dashed horizontal line* shows the maximum accumulated richness for Grade A. The vertical lines** show the number of observations to reach Grade A's maximum accumulated richness in Grade A (left vertical line) and in Grade D (right vertical line). The difference in observations between redlined (i.e., grade D) and greenlined (i.e., grade A) and neighborhoods is shown as a delta value.

*Horizontal lines (y): native = 506; nonnative = 458

**Vertical lines (x; grade A, grade D): native = 4607, 5228, nonnative = 2941, 3632

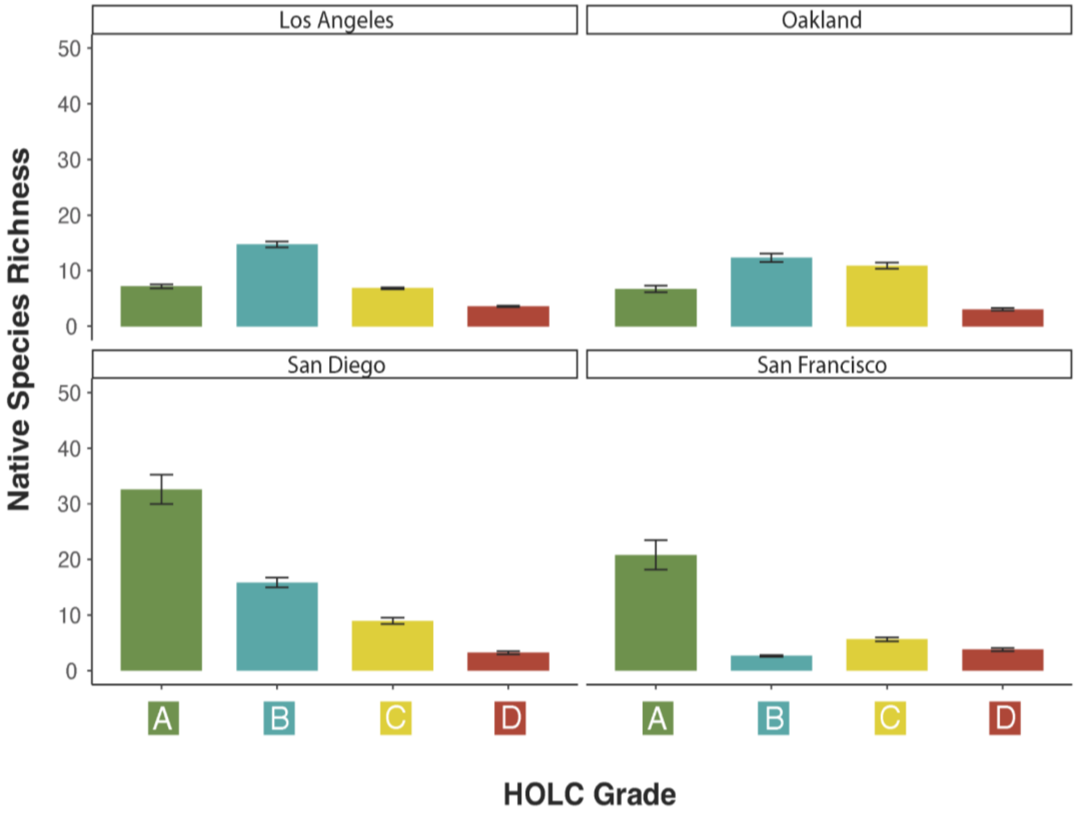

Figure S3. City-level differences in native species richness across HOLC grades. The relationship between HOLC grade and native species richness for Los Angeles (top left), Oakland (top right), (C) San Diego (bottom left), and San Francisco (bottom right). Bars represent the mean, and whiskers represent 2.5 and 97.5% confidence intervals. All pair-wise comparisons are significant except grades A and C in Los Angeles.

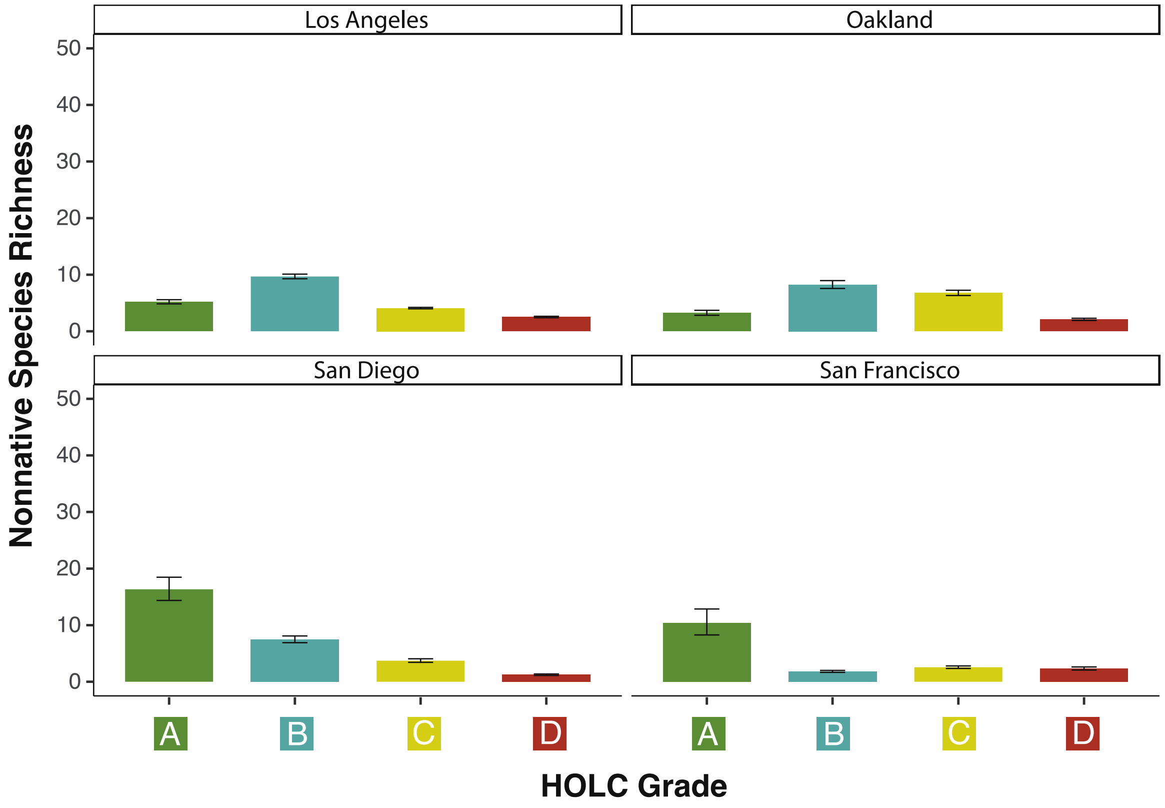

Figure S4. City-level differences in nonnative species richness. The relationship between HOLC grade and nonnative species richness for Los Angeles (top left), Oakland (top right), (C) San Diego (bottom left), and San Francisco (bottom right). Bars represent the mean, and whiskers represent 2.5 and 97.5% confidence intervals. All pair-wise comparisons are significant except grades C and D in San Francisco.

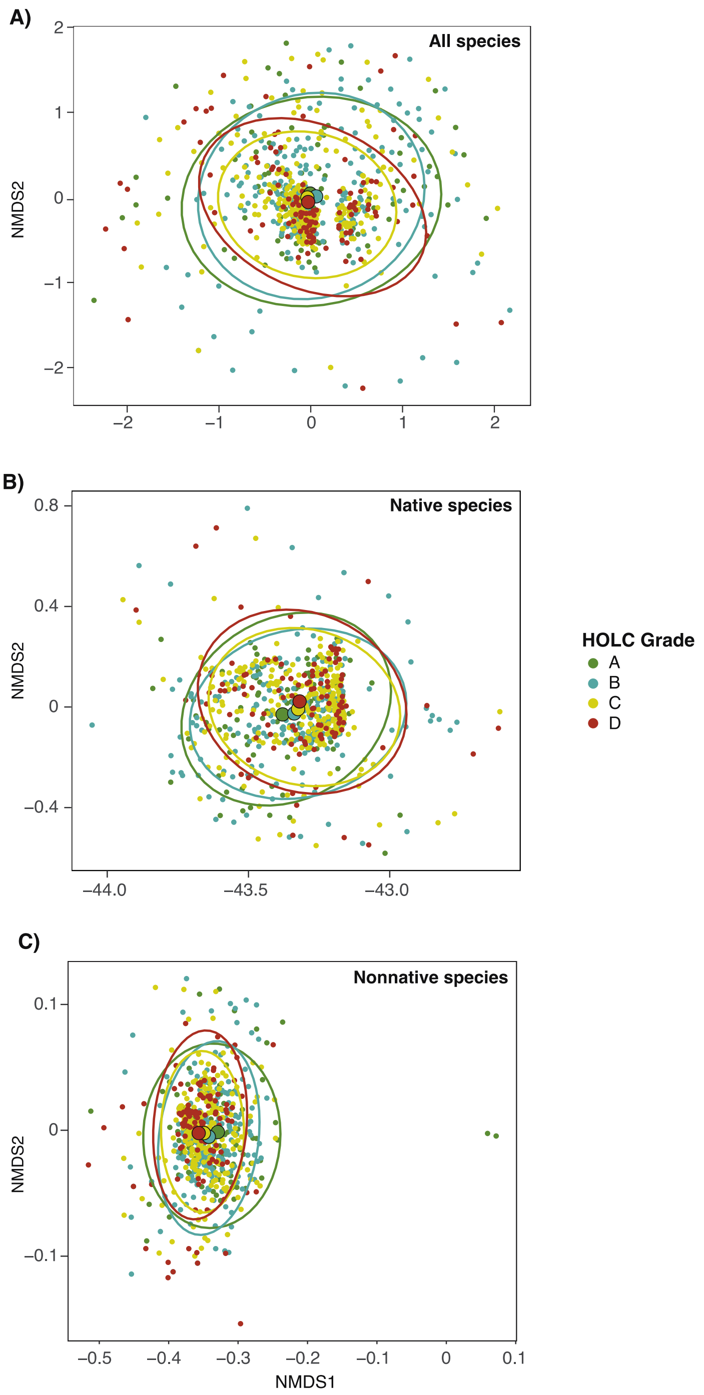

Figure S5. HOLC grade beta diversity**.** Non-metric multidimensional scaling (NMDS) for β-diversity (Jaccard's metric) among HOLC grades for (A) all species, (B) native species, and (C) nonnative species. Each dot represents a neighborhood within a city and ellipses encompass 95% data points. No overlap between ellipses suggests that HOLC grades have distinct beta diversity patterns and strong dissimilarity in species assemblage. Substantial overlap in ellipses suggests that beta diversity between HOLC grades is more similar to each other and there is strong similarity in species assemblage.

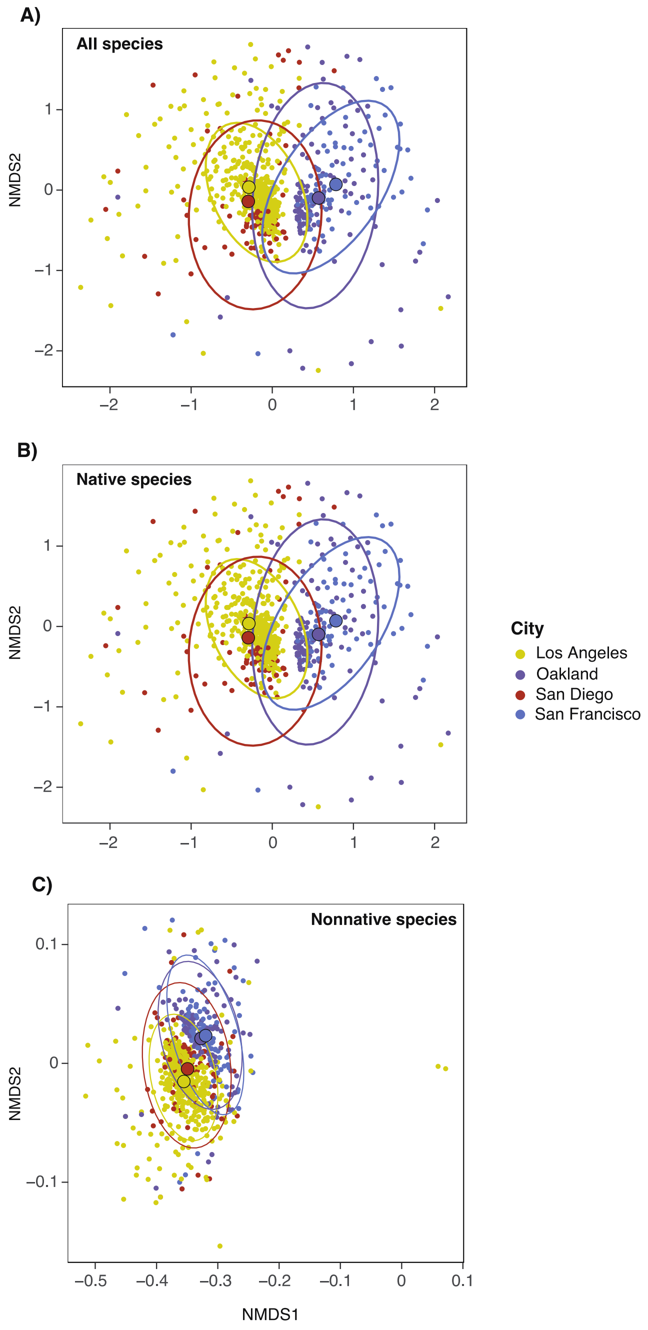

Figure S6. Beta diversity per city. Non-metric multidimensional scaling (NMDS) for β-diversity (Jaccard's metric) among cities for (A) all species, (B) native species, and (C) nonnative species. Each dot represents a neighborhood within a city and ellipses encompass 95% data points. No overlap between ellipses suggests that cities have distinct beta diversity patterns and strong dissimilarity in species assemblage. Substantial overlap in ellipses suggests that beta diversity between cities is more similar to each other and there is strong similarity in species assemblage. *Note: Outlier points removed for native (2 points) and nonnative species (2 points) in Los Angeles as well as nonnative species (1 point) in Oakland to assist in visualization.*

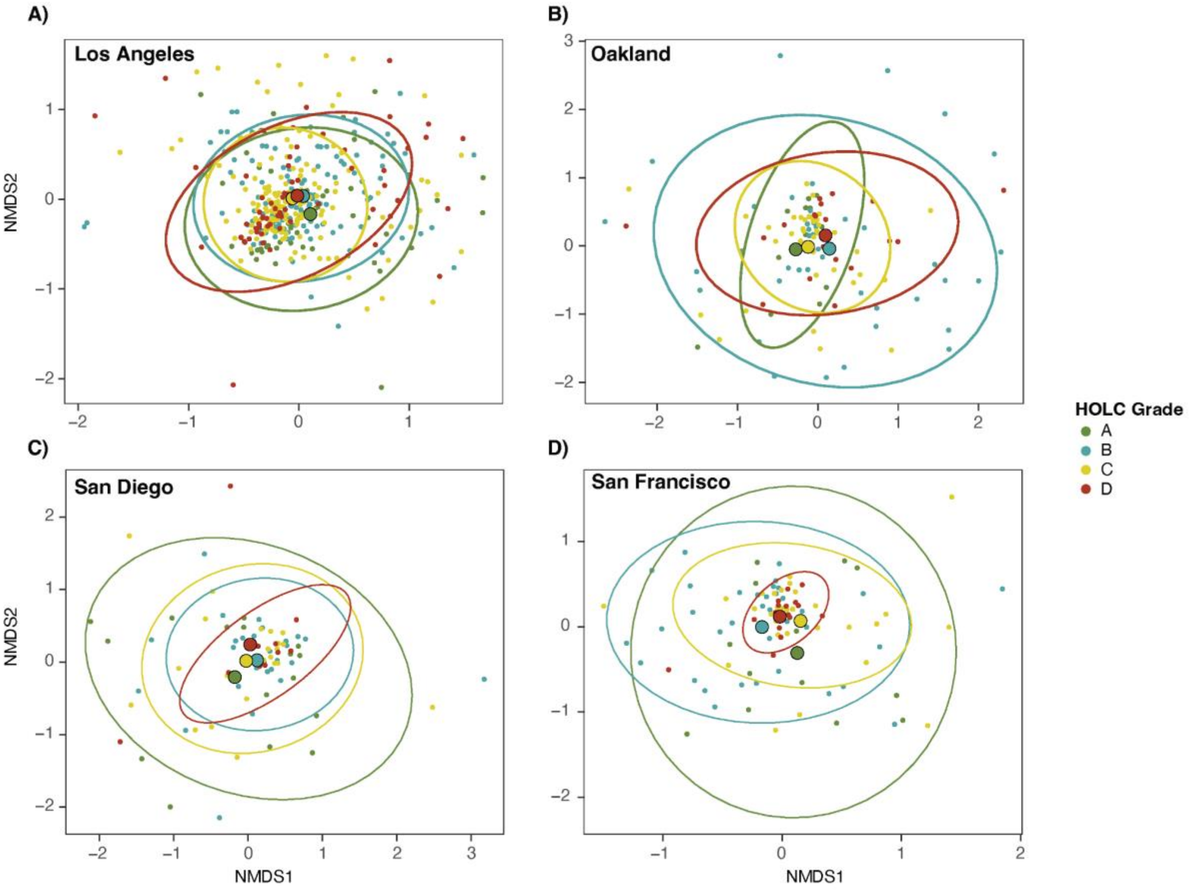

Figure S7. HOLC grade native beta diversity by city. Non-metric multidimensional scaling (NMDS) for native β-diversity (Jaccard's metric) among HOLC grades in (A) Los Angeles, (B) Oakland, (C) San Diego, and (D) San Francisco. Each dot represents a neighborhood within a city and ellipses encompass 95% data points. No overlap between ellipses suggests that HOLC grades have distinct beta diversity patterns and strong dissimilarity in native species assemblage. Substantial overlap in ellipses suggests that beta diversity between HOLC grades is more similar to each other and there is strong similarity in native species assemblage. *Note: Outlier points removed for Los Angeles to assist in visualization (2 points in grade D).*

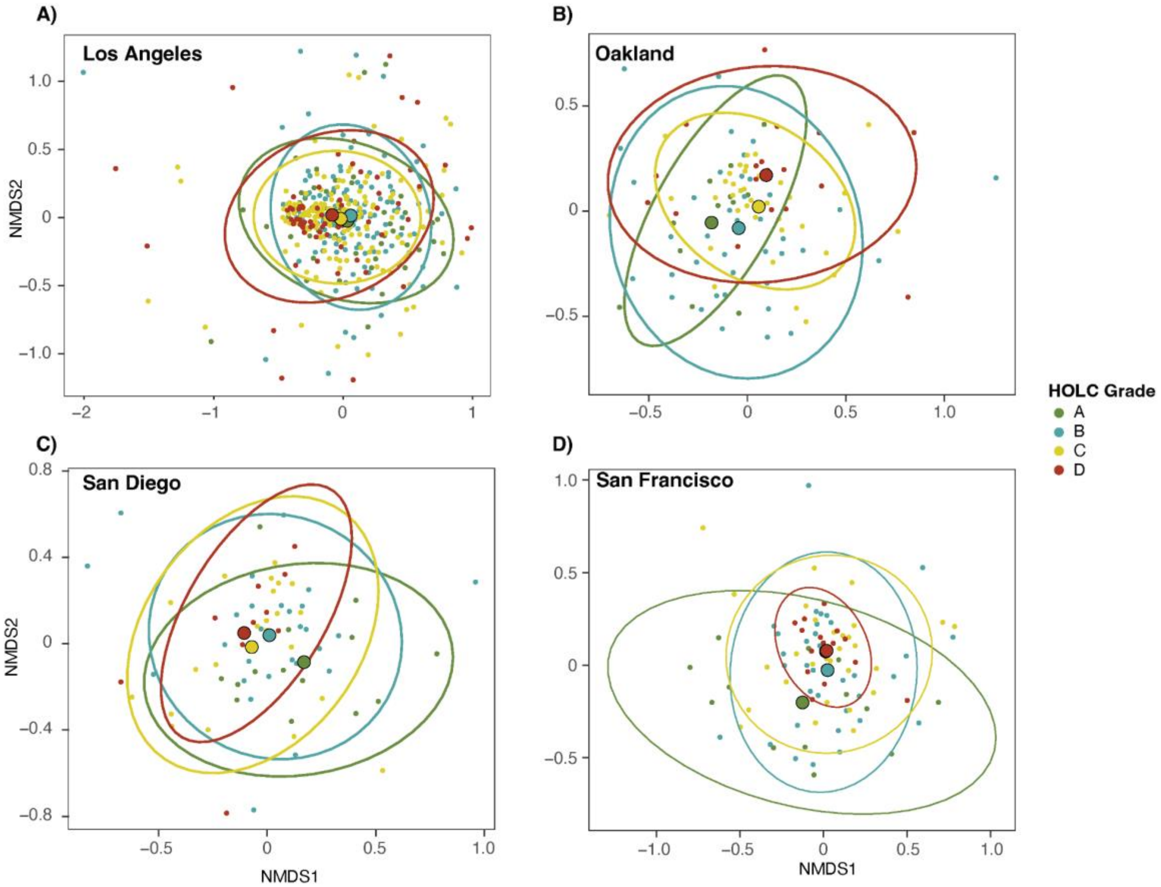

Figure S8. HOLC grade nonnative beta diversity by city. Non-metric multidimensional scaling (NMDS) for nonnative β-diversity (Jaccard's metric) among HOLC grades in (A) Los Angeles, (B) Oakland, (C) San Diego, and (D) San Francisco. Each dot represents a neighborhood within a city and ellipses encompass 95% data points. No overlap between ellipses suggests that HOLC grades have distinct beta diversity patterns and strong dissimilarity in nonnative species assemblage. Substantial overlap in ellipses suggests that beta diversity between HOLC grades is more similar to each other and there is strong similarity in nonnative species assemblage. *Note: Outlier points removed for Los Angeles (2 points in grade D) and Oakland (1 point in grade C) to assist in visualization*.

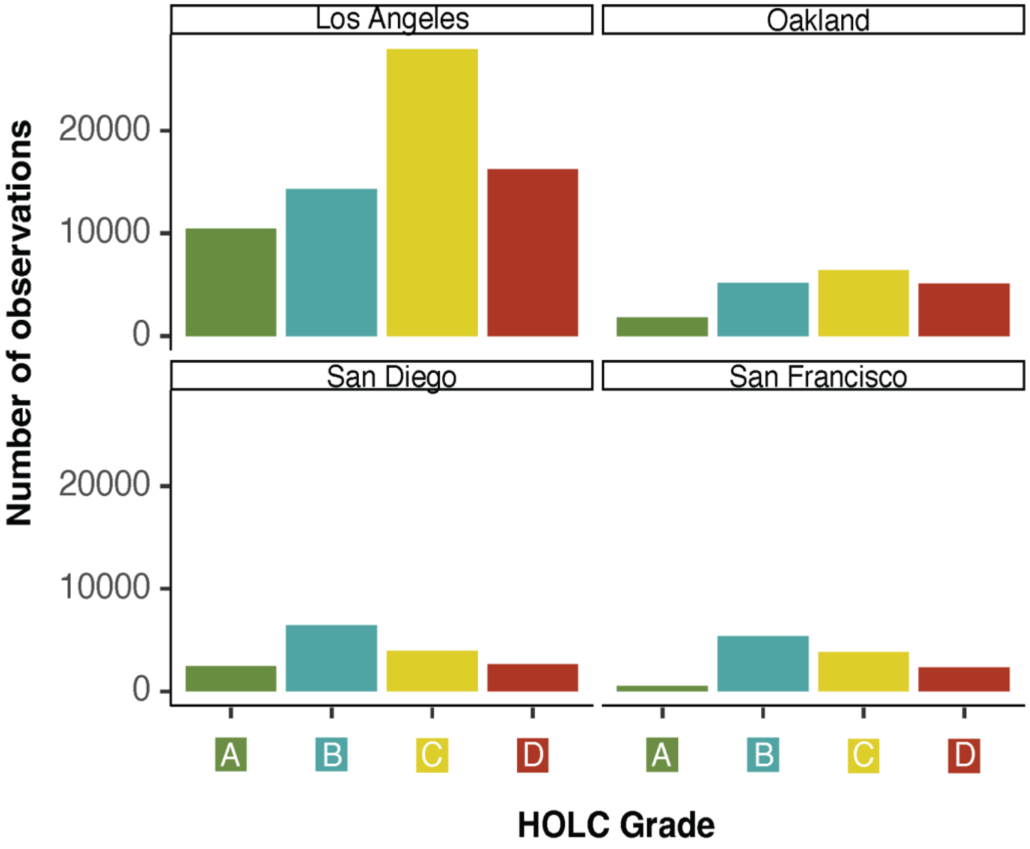

Figure S9. iNaturalist observations across HOLC grades per city. iNaturalist observations for Los Angeles (top left), Oakland (top right), (C) San Diego (bottom left), and San Francisco (bottom right) for each HOLC grade.

Supporting Information 3: Tables

Table S1. Overall clade species richness.

Clade	HOLC Grade	Mean Richness	Mean Native Richness	Mean Nonnative Richness
Mammal	A	1.29 (0.24, 3.83)	0.69 (0.17, 1.76)	1.03 (0.22, 3.01)
Mammal	B	1.58 (0.26, 5.88)	0.81 (0.16, 2.6)	1.52 (0.29, 4.52)
Mammal	C	1.07 (0.18, 4)	0.74 (0.15, 2.23)	0.63 (0.1, 2.69)
Mammal	D	0.53 (0.08, 2.31)	0.29 (0.06, 1.04)	0.39 (0.06, 1.69)
Bird	A	6.91 (1.22, 20.96)	5.96 (1.06, 17.82)	1.05 (0.18, 3.4)
Bird	B	4.88 (0.55, 31.72)	4.03 (0.46, 26.14)	1.07 (0.12, 6.06)
Bird	C	4.15 (0.59, 19.14)	3.49 (0.51, 15.91)	0.85 (0.12, 4.02)
Bird	D	1.75 (0.27, 7.36)	1.46 (0.23, 6.01)	0.36 (0.05, 1.76)
Insect	A	8.91 (1.3, 29.65)	4.5 (0.63, 15.5)	4.7 (0.71, 15.07)
Insect	B	6.27 (0.67, 38.31)	3.17 (0.33, 19.47)	3.39 (0.37, 21.27)
Insect	C	4.88 (0.54, 28.77)	2.52 (0.26, 15.62)	2.57 (0.29, 14.54)
Insect	D	2.58 (0.3, 15.25)	1.29 (0.14, 7.84)	1.4 (0.17, 7.87)
Arachnid	A	1.72 (0.25, 5.83)	0.63 (0.09, 2.43)	1.5 (0.32, 4.05)
Arachnid	B	1.74 (0.18, 10.29)	0.85 (0.09, 5.03)	1.56 (0.23, 6.6)
Arachnid	C	1.17 (0.12, 7.39)	0.55 (0.05, 3.85)	1 (0.15, 4.67)
Arachnid	D	0.56 (0.06, 3.97)	0.25 (0.02, 1.96)	0.52 (0.07, 2.96)
Reptile	A	1.37 (0.24, 4.51)	1.42 (0.24, 4.59)	0.36 (0.1, 1.18)
Reptile	B	1.41 (0.18, 6.63)	1.45 (0.19, 6.86)	0.97 (0.16, 3.79)

Reptile	C	0.79 (0.1, 4.63)	0.8 (0.1, 4.69)	0.57 (0.13, 1.91)
Reptile	D	0.4 (0.05, 2.88)	0.43 (0.05, 3.19)	0.16 (0.04, 0.65)
Amphibian	A	0.88 (0.49, 1.53)	0.84 (0.42, 1.56)	0.82 (0.21, 2.99)
Amphibian	B	0.9 (0.28, 2.64)	0.9 (0.25, 2.83)	1.4 (0.22, 8.72)
Amphibian	C	0.5 (0.24, 0.86)	0.5 (0.2, 0.97)	0.47 (0.1, 2.42)
Amphibian	D	0.23 (0.1, 0.52)	0.23 (0.09, 0.55)	0.16 (0.03, 0.99)

Species richness for each clade is shown for all cities per HOLC grade (grades A = “best” and “greenlined”, B, C, and D = “hazardous” and “redlined”). We show overall species richness, native species richness, and nonnative species richness with mean and 95% credible intervals.

Table S2. City-level mammal species richness.

City	HOLC Grade	Mean Richness	Mean Native Richness	Mean Nonnative Richness
Los Angeles	A	4.7 (4.42, 4.99)	2.34 (2.14, 2.56)	2.63 (2.41, 2.87)
Los Angeles	B	8.75 (8.42, 9.09)	4.38 (4.16, 4.62)	4.89 (4.62, 5.16)
Los Angeles	C	4.35 (4.24, 4.48)	2.32 (2.23, 2.41)	2.27 (2.18, 2.36)
Los Angeles	D	2.98 (2.85, 3.12)	1.52 (1.43, 1.62)	1.62 (1.52, 1.73)
Oakland	A	5.17 (4.64, 5.77)	2.24 (1.91, 2.6)	2.43 (2.05, 2.87)
Oakland	B	9.83 (9.16, 10.52)	3.73 (3.38, 4.11)	5.32 (4.82, 5.86)
Oakland	C	9.77 (9.22, 10.34)	4.27 (3.94, 4.61)	4.53 (4.16, 4.91)
Oakland	D	2.95 (2.72, 3.18)	1.29 (1.15, 1.43)	1.36 (1.22, 1.52)
San Diego	A	21.71 (19.54, 24.1)	12.81 (11.05, 14.76)	8.95 (7.67, 10.4)
San Diego	B	8.46 (7.91, 9.04)	4.88 (4.44, 5.34)	3.68 (3.34, 4.04)
San Diego	C	4.64 (4.31, 4.99)	2.72 (2.45, 3)	2 (1.79, 2.22)
San Diego	D	1.77 (1.61, 1.94)	1.09 (0.96, 1.24)	0.71 (0.62, 0.81)
San Francisco	A	16.18 (13.5, 19.2)	10.3 (7.94, 13.22)	8.01 (6.13, 10.18)
San Francisco	B	2.83 (2.6, 3.07)	2.11 (1.87, 2.36)	1.31 (1.17, 1.47)

San Francisco	C	3.53 (3.27, 3.81)	2.31 (2.07, 2.58)	1.84 (1.65, 2.03)
San Francisco	D	2.91 (2.65, 3.2)	1.64 (1.43, 1.86)	1.74 (1.51, 1.98)

Mammalian species richness is shown for each city per HOLC grade (grades A = "best" and "greenlined", B, C, and D = "hazardous" and "redlined"). We show overall species richness, native species richness, and nonnative species richness with mean and 95% credible intervals.

147 **Table S3.** City-level bird species richness across HOLC grades.

City	HOLC Grade	Mean Richness	Mean Native Richness	Mean Nonnative Richness
Los Angeles	A	3.79 (3.53, 4.07)	3.26 (3.02, 3.5)	0.88 (0.73, 1.05)
Los Angeles	B	8.68 (8.3, 9.07)	7.4 (7.04, 7.76)	2.06 (1.85, 2.29)
Los Angeles	C	3.46 (3.35, 3.58)	3.07 (2.96, 3.19)	0.71 (0.66, 0.77)
Los Angeles	D	1.57 (1.49, 1.65)	1.37 (1.3, 1.45)	0.34 (0.3, 0.39)
Oakland	A	3.81 (3.35, 4.33)	3.63 (3.17, 4.16)	0.36 (0.24, 0.53)
Oakland	B	7.19 (6.58, 7.84)	6.26 (5.71, 6.86)	1.03 (0.76, 1.38)
Oakland	C	5.51 (5.15, 5.89)	4.78 (4.45, 5.14)	0.81 (0.65, 1)
Oakland	D	1.35 (1.23, 1.48)	1.14 (1.03, 1.26)	0.25 (0.19, 0.32)
San Diego	A	15.81 (14.23, 17.51)	15.47 (13.84, 17.22)	1.71 (1.19, 2.37)
San Diego	B	7.94 (7.37, 8.55)	7.64 (7.05, 8.26)	1.12 (0.91, 1.37)
San Diego	C	4.85 (4.43, 5.3)	4.7 (4.25, 5.17)	0.67 (0.54, 0.83)
San Diego	D	1.81 (1.62, 2.02)	1.82 (1.61, 2.05)	0.21 (0.15, 0.27)
San Francisco	A	11.16 (9.41, 13.11)	9.45 (7.92, 11.2)	1.33 (0.63, 2.67)
San Francisco	B	1.21 (1.12, 1.31)	0.98 (0.9, 1.06)	0.34 (0.26, 0.43)
San Francisco	C	3.52 (3.23, 3.82)	2.9 (2.65, 3.16)	0.78 (0.6, 1)
San Francisco	D	2.36 (2.12, 2.62)	1.95 (1.73, 2.18)	0.46 (0.34, 0.6)

Avian species richness is shown for each city per HOLC grade (grades A = “best” and “greenlined”, B, C, and D = “hazardous” and “redlined”). We show overall species richness, native species richness, and nonnative species richness with mean and 95% credible intervals.

Table S4. City-level insect species richness.

City	HOLC Grade	Mean Richness	Mean Native Richness	Mean Nonnative Richness
Los Angeles	A	4.7 (4.42, 4.99)	2.34 (2.14, 2.56)	2.63 (2.41, 2.87)
Los Angeles	B	8.75 (8.42, 9.09)	4.38 (4.16, 4.62)	4.89 (4.62, 5.16)
Los Angeles	C	4.35 (4.24, 4.48)	2.32 (2.23, 2.41)	2.27 (2.18, 2.36)
Los Angeles	D	2.98 (2.85, 3.12)	1.52 (1.43, 1.62)	1.62 (1.52, 1.73)
Oakland	A	5.17 (4.64, 5.77)	2.24 (1.91, 2.6)	2.43 (2.05, 2.87)
Oakland	B	9.83 (9.16, 10.52)	3.73 (3.38, 4.11)	5.32 (4.82, 5.86)
Oakland	C	9.77 (9.22, 10.34)	4.27 (3.94, 4.61)	4.53 (4.16, 4.91)
Oakland	D	2.95 (2.72, 3.18)	1.29 (1.15, 1.43)	1.36 (1.22, 1.52)
San Diego	A	21.71 (19.54, 24.1)	12.81 (11.05, 14.76)	8.95 (7.67, 10.4)
San Diego	B	8.46 (7.91, 9.04)	4.88 (4.44, 5.34)	3.68 (3.34, 4.04)
San Diego	C	4.64 (4.31, 4.99)	2.72 (2.45, 3)	2 (1.79, 2.22)
San Diego	D	1.77 (1.61, 1.94)	1.09 (0.96, 1.24)	0.71 (0.62, 0.81)
San Francisco	A	16.18 (13.5, 19.2)	10.3 (7.94, 13.22)	8.01 (6.13, 10.18)
San Francisco	B	2.83 (2.6, 3.07)	2.11 (1.87, 2.36)	1.31 (1.17, 1.47)

San Francisco	C	3.53 (3.27, 3.81)	2.31 (2.07, 2.58)	1.84 (1.65, 2.03)
San Francisco	D	2.91 (2.65, 3.2)	1.64 (1.43, 1.86)	1.74 (1.51, 1.98)

Insect species richness is shown for each city per HOLC grade (grades A = “best” and “greenlined”, B, C, and D = “hazardous” and “redlined”). We show overall species richness, native species richness, and nonnative species richness with mean and 95% credible intervals.

Table S5. City-level arachnid species richness.

City	HOLC Grade	Mean Richness	Mean Native Richness	Mean Nonnative Richness
Los Angeles	A	1.35 (1.15, 1.58)	0.41 (0.31, 0.54)	1.48 (1.23, 1.77)
Los Angeles	B	2.27 (2.07, 2.48)	0.85 (0.72, 1)	2.14 (1.92, 2.37)
Los Angeles	C	1.02 (0.95, 1.09)	0.5 (0.45, 0.57)	0.83 (0.76, 0.9)
Los Angeles	D	0.68 (0.61, 0.76)	0.32 (0.26, 0.39)	0.57 (0.5, 0.65)
Oakland	A	0.66 (0.5, 0.87)	0.18 (0.12, 0.28)	0.65 (0.45, 0.92)
Oakland	B	2.57 (2.1, 3.11)	0.79 (0.57, 1.07)	1.94 (1.48, 2.49)
Oakland	C	2.39 (2.04, 2.77)	0.85 (0.65, 1.08)	1.63 (1.34, 1.97)
Oakland	D	0.78 (0.63, 0.96)	0.27 (0.2, 0.37)	0.58 (0.43, 0.77)
San Diego	A	4.09 (2.92, 5.59)	2.27 (1.26, 3.74)	3.07 (1.89, 4.69)
San Diego	B	1.91 (1.56, 2.31)	0.99 (0.71, 1.34)	1.95 (1.51, 2.48)
San Diego	C	1.06 (0.87, 1.28)	0.83 (0.6, 1.12)	0.83 (0.64, 1.06)
San Diego	D	0.21 (0.16, 0.26)	0.14 (0.1, 0.2)	0.18 (0.13, 0.24)
San Francisco	A	2.25 (1.25, 3.65)	1.51 (0.7, 2.88)	1.42 (0.59, 3.19)
San Francisco	B	0.88 (0.71, 1.09)	1.54 (1.06, 2.15)	0.62 (0.47, 0.81)
San Francisco	C	0.66 (0.54, 0.8)	0.45 (0.32, 0.61)	0.68 (0.53, 0.87)
San Francisco	D	0.56 (0.44, 0.72)	0.34 (0.24, 0.48)	0.73 (0.5, 1.02)

Arachnid species richness is shown for each city per HOLC grade (grades A = “best” and “greenlined”, B, C, and D = “hazardous” and “redlined”). We show overall species richness, native species richness, and nonnative species richness with mean and 95% credible intervals.

Table S6. City-level reptile species richness.

City	HOLC Grade	Mean Richness	Mean Native Richness	Mean Nonnative Richness
Los Angeles	A	0.96 (0.79, 1.16)	0.88 (0.71, 1.07)	0.32 (0.17, 0.62)
Los Angeles	B	1.65 (1.46, 1.86)	1.53 (1.34, 1.73)	0.68 (0.44, 0.99)
Los Angeles	C	0.6 (0.54, 0.66)	0.53 (0.48, 0.59)	0.44 (0.32, 0.58)
Los Angeles	D	0.32 (0.27, 0.37)	0.31 (0.26, 0.37)	0.12 (0.09, 0.17)
Oakland	A	0.21 (0.14, 0.31)	0.22 (0.14, 0.33)	0.22 (0.08, 0.58)
Oakland	B	0.43 (0.3, 0.61)	0.45 (0.31, 0.64)	0.47 (0.03, 1.89)
Oakland	C	0.45 (0.33, 0.6)	0.43 (0.31, 0.59)	0.5 (0.19, 1.39)
Oakland	D	0.26 (0.15, 0.43)	0.32 (0.18, 0.53)	0.11 (0.03, 0.43)
San Diego	A	3.62 (2.49, 5.1)	3.51 (2.38, 4.98)	0.32 (0.12, 1.05)
San Diego	B	1.46 (1.13, 1.84)	1.39 (1.07, 1.77)	1.33 (0.41, 4.58)
San Diego	C	0.54 (0.4, 0.7)	0.48 (0.35, 0.64)	0.41 (0.2, 0.77)
San Diego	D	0.18 (0.14, 0.24)	0.16 (0.11, 0.21)	0.11 (0.06, 0.18)
San Francisco	A	1.19 (0.47, 2.74)	1.06 (0.42, 2.43)	0.41 (0.1, 1.63)
San Francisco	B	1 (0.52, 1.74)	0.86 (0.42, 1.57)	1.41 (0.32, 4.81)
San Francisco	C	0.61 (0.33, 1.02)	0.58 (0.29, 1.05)	0.66 (0.2, 1.69)
San Francisco	D	0.25 (0.16, 0.38)	0.22 (0.13, 0.34)	0.24 (0.06, 1.08)

Reptile species richness is shown for each city per HOLC grade (grades A = “best” and “greenlined”, B, C, and D = “hazardous” and “redlined”). We show overall species richness, native species richness, and nonnative species richness with mean and 95% credible intervals.

Table S7. City-level amphibian species richness.

City	HOLC Grade	Mean Richness	Mean Native Richness	Mean Nonnative Richness
Los Angeles	A	0.85 (0.59, 1.21)	0.79 (0.51, 1.17)	0.79 (0.34, 1.76)
Los Angeles	B	1.53 (1.06, 2.14)	1.63 (1.07, 2.38)	1.05 (0.49, 1.95)
Los Angeles	C	0.5 (0.4, 0.63)	0.56 (0.41, 0.75)	0.34 (0.2, 0.54)
Los Angeles	D	0.21 (0.15, 0.29)	0.23 (0.15, 0.35)	0.15 (0.08, 0.27)
Oakland	A	1.36 (0.91, 2.01)	1.34 (0.85, 2.08)	1.27 (0.3, 6.19)
Oakland	B	1.91 (1.42, 2.52)	2 (1.48, 2.66)	2.13 (0.31, 9.82)
Oakland	C	0.81 (0.63, 1.02)	0.83 (0.65, 1.06)	0.81 (0.22, 2.71)
Oakland	D	0.29 (0.2, 0.41)	0.3 (0.2, 0.42)	0.25 (0.02, 4.22)
San Diego	A	0.68 (0.41, 1.24)	0.68 (0.38, 1.4)	1.22 (0.3, 5.89)
San Diego	B	0.79 (0.41, 1.37)	0.84 (0.44, 1.45)	2.03 (0.11, 55.02)
San Diego	C	0.35 (0.18, 0.53)	0.34 (0.16, 0.54)	0.88 (0.16, 6.67)
San Diego	D	0.13 (0.06, 0.23)	0.14 (0.06, 0.25)	0.22 (0.04, 0.69)
San Francisco	A	0.56 (0.32, 1.26)	0.55 (0.3, 1.4)	0.04 (0.01, 0.68)
San Francisco	B	0.21 (0.15, 0.3)	0.21 (0.14, 0.29)	0.12 (0.01, 2.47)
San Francisco	C	0.31 (0.21, 0.49)	0.32 (0.21, 0.5)	0.02 (0, 0.77)
San Francisco	D	0.22 (0.13, 0.37)	0.23 (0.13, 0.37)	0.01 (0, 0.27)

Amphibian species richness is shown for each city per HOLC grade (grades A = “best” and “greenlined”, B, C, and D = “hazardous” and “redlined”). We show overall species richness, native species richness, and nonnative species richness with mean and 95% credible intervals.

Table S8. Beta diversity pair-wise comparisons.

Species	City	A-B	A-C	A-D	B-C	B-D	C-D
All	All	**$p < 0.05$**	**$p < 0.001$**	**$p < 0.001$**	**$p < 0.001$**	**$p < 0.001$**	**$p < 0.01$**
All	Los Angeles	**$p < 0.01$**	**$p < 0.001$**	**$p < 0.01$**	**$p < 0.01$**	**$p < 0.001$**	**$p < 0.05$**
All	Oakland	$p = 0.068$	$p = 0.053$	**$p < 0.05$**	**$p < 0.05$**	**$p < 0.05$**	$p = 0.221$
All	San Diego	$p = 0.508$	**$p < 0.05$**	**$p < 0.05$**	$p = 0.761$	$p = 0.508$	$p = 0.761$
All	San Francisco	$p = 0.0922$	**$p < 0.01$**	**$p < 0.001$**	$p = 0.0708$	**$p < 0.001$**	**$p < 0.01$**
Native	All	**$p < 0.01$**	**$p < 0.001$**	**$p < 0.001$**	**$p < 0.001$**	**$p < 0.001$**	**$p < 0.05$**
Native	Los Angeles	**$p < 0.01$**	**$p < 0.001$**	**$p < 0.01$**	**$p < 0.01$**	**$p < 0.01$**	$p = 0.0707$
Native	Oakland	$p = 0.073$	**$p < 0.05$**	**$p < 0.05$**	**$p < 0.05$**	**$p < 0.05$**	$p = 0.363$
Native	San Diego	$p = 0.619$	$p = 0.091$	$p = 0.091$	$p = 0.886$	$p = 0.696$	$p = 0.696$
Native	San Francisco	$p = 0.2626$	**$p < 0.05$**	**$p < 0.001$**	$p = 0.0557$	**$p < 0.001$**	**$p < 0.05$**
Nonnative	All	$p = 0.26967$	**$p < 0.001$**	**$p < 0.001$**	**$p < 0.001$**	**$p < 0.001$**	**$p < 0.01$**
Nonnative	Los Angeles	$p = 0.0972$	**$p < 0.001$**	**$p < 0.01$**	**$p < 0.01$**	**$p < 0.001$**	**$p < 0.01$**
Nonnative	Oakland	$p = 0.326$	$p = 0.326$	**$p < 0.05$**	$p = 0.089$	**$p < 0.05$**	$p = 0.197$
Nonnative	San Diego	$p = 0.630$	$p = 0.071$	**$p < 0.05$**	$p = 0.630$	$p = 0.376$	$p = 0.936$
Nonnative	San Francisco	**$p < 0.05$**	**$p < 0.01$**	**$p < 0.001$**	$p = 0.3210$	**$p < 0.01$**	**$p < 0.05$**

Pair-wise comparisons for beta diversity via PERMANOVA for each city is shown for all species, native species, and nonnative species. We used a PERMANOVA with 10000 permutations to determine which specific HOLC grade dyads (e.g., A vs. C, A vs. D, etc.)

significantly differed in species assemblage with a Benjamin-Hochberg correction. Significant comparisons are bolded.
